# Effects of 92% oxygen administration on cognitive performance and physiological changes of intellectually and developmentally disabled people

**DOI:** 10.1186/s40101-015-0043-9

**Published:** 2015-02-20

**Authors:** Hyung-Sik Kim, Mi-Hyun Choi, Ji-Hye Baek, Sung-Jun Park, Jung-Chul Lee, Ul-Ho Jeong, Sung-Phil Kim, Hyun-Jun Kim, Young Chil Choi, Dae-Woon Lim, Soon-Cheol Chung

**Affiliations:** Department of Biomedical Engineering, BK21+ Research Institute of Biomedical Engineering, College of Biomedical & Health Science, Konkuk University, 268 Chungwon-daero, Chungju, Chungbuk-do 380-701 South Korea; Department of Design Human Engineering, Ulsan National Institute of Science and Technology, 50 UNIST-gil, Ulsan, 689-798 South Korea; Department of Obstetrics & Gynecology, Konkuk University, 268 Chungwon-daero, Chungju, Chungbuk-do 380-701 South Korea; Department of Radiology, School of Medicine, Konkuk University, 268 Chungwon-daero, Chungju, Chungbuk-do 380-701 South Korea; Department of Information & Communication Engineering, Dongguk University, 30 Pildong-ro, Seoul, 100-715 South Korea

**Keywords:** Oxygen administration, Cognitive performance, Blood oxygen saturation, Heart rate, Intellectual and developmental disability

## Abstract

**Background:**

The present study addressed how 92% oxygen administration affects cognitive performance, blood oxygen saturation (SpO_2_), and heart rate (HR) of intellectually and developmentally disabled people.

**Methods:**

Seven males (28.9 ± 1.8 years) and seven females (34.4 ± 8.3 years) with intellectual and developmental disabilities (disabled level 2.1 ± 0.5) completed an experiment consisting a 0-back task with normal air (21% oxygen) administered in one run and hyperoxic air (92% oxygen) administered in the other run. The experimental sequence in each run consisted of a 1-min adaptation phase, 2-min control phase, and 2-min 0-back task phase, where SpO_2_ and HR were gauged for each phase.

**Results:**

The administration of 92% oxygen increased 0-back task performance of intellectually and developmentally disabled people, in association with increased SpO_2_ and decreased HR. Our results demonstrate that sufficient oxygen supply subserving cognitive functions, even as a short-term effect, could increase cognitive ability for the intellectually and developmentally disabled people.

**Conclusions:**

It is concluded that enriched oxygen can positively affect, at least in the short-term, the working memory of those with intellectual and developmental disability.

## Background

Intellectually and developmentally disabled people have difficulties in their social and personal lives as a consequence of insufficient or incomplete intellectual development due to the permanent retardation of physical and intellectual growth [1]. One of the most profound characteristics of those with intellectual and developmental disability is cognitive deficiency [1]. The cognitive impairment impairs information processing, problem solving, and attentive concentration [2-6]. Furthermore, the deficient capability in organizing informational units upon the simultaneous reception of myriad of information translates to memory deficiency [2,4]. Short-term and working memory are especially affected, rendering subjects forgetful of recently acquired information or learned tasks, as well as applying what they have learned in their daily lives [7] and in recalling information stored in memory [3].

The administration of high-concentration oxygen increases memory capability of healthy young and elderly individuals [8-12]. As well, exposure to highly concentrated oxygen positively affects *n*-back tasks, visuospatial perception, use of verbs, and efficiency in addition performance of healthy young adults [13-19]. Recently, external oxygen administration has been shown to have positive effects on visual matching task performance of intellectually and developmentally disabled individuals [20].

The administration of high-concentration oxygen also leads to cognitive enhancement, as revealed by increases in accuracy [8,10,11,13-18] and/or decreases in response time [8-12,14,16,19,20] during cognitive tasks. Physiological responses such as increased blood oxygen saturation (SpO_2_) as well as decreased heart rate (HR) have supported the contribution of high-concentration oxygen administration to cognitive performance [8-12,14,16-20].

Many studies with a variety of cognitive tasks were performed using various verification methods to clarify how the administration of high-concentration oxygen enhances cognitive capability of normal people [8-19]. However, similar examinations with intellectually and developmentally disabled individuals have involved only one study using a visual matching task [20]. Further studies employing a variety of cognitive tasks as well as verification methods are needed in this population.

In this study, we investigated the effect of the administration of high-concentration oxygen on the memory function of individuals with intellectual and developmental disability. SpO_2_ and HR were measured, and the 0-back task was used as a cognitive task to focus on short-term memory, which is one of the most critical cognitive defects of those with intellectual and developmental disability.

## Methods

The participants of this study were workers diagnosed by qualified psychiatrists as intellectually and developmentally disabled. The workers were all from a protective workshop of a community welfare foundation. Seven males (28.9 ± 1.8 years of age) and seven females (34.4 ± 8.3 years of age) with an assessed disability level of 2.1 ± 0.5 participated. Disability level was determined by a psychiatrist based on Diagnostic and Statistical Manual of Mental Disorders - 4th edition (DSM-IV) [21]. None of the participants reported having a periphery vascular flow system or respiratory system abnormality. The overall procedures and purposes of the experiments were explained to all the subjects and their guardians, and their guardians’ consent was.

The protocol for the research project has been approved by Institutional Review Committee of Konkuk University within which the work was undertaken and that it conforms to the provisions of the Declaration of Helsinki.

The oxygen supply equipment (OXUS Co., Seoul, Korea) developed for this study consistently provided either 21% (21% O_2_, 78% N_2_, and 1% Ar) or 92% (92% O_2_, 7% N_2_, and 1% Ar) oxygen in the air at a constant rate of 5 L/min. Oxygen was supplied to each subject through a mask to ensure a steady flow of oxygen with a constant concentration level.

The experiment was composed of two separate runs of a 0-back task. One run was conducted in the presence of 21% oxygen and the other with a 92% oxygen level. Every subject completed two runs where the order of runs was counterbalanced between 21% and 92% conditions. The second run started 1 h after the first one. Each run consisted of three phases: adaptation (1-min adaptation period after starting the oxygen administration), control (2-min stabilization period prior to the task), and 0-back task (2 min). During 2-min 0-back task phase, 20 numbers comprising one of five single-digit numbers (0, 1, 2, 3, or 4) were presented to the subject at 6-s intervals. The use of five single-digit numbers was in consideration of the intellectual level of the subjects. Each subject was asked to press the button when a certain number (0) was displayed. The ratio of the target number to the total numbers was 6:20. The 0-back tasks were presented on a monitor using E-prime (Psychology Software Tools, Sharpsburg, PA, USA). Prior to the start of the experiment, the subject was carefully instructed regarding the procedures and shown how to complete the task in the experiment.

The accuracy rate (%) was determined as (number of correct answers ÷ total number) × 100. The response time in milliseconds (ms) for each facet of the 0-back test for each oxygen concentration was determined. We used a paired *t*-test (PASW ver. 18.0; SPSS, Chicago, IL, USA) to evaluate the differences in the accuracy rate and the response time between two oxygen concentration conditions.

An 8600 Series pulse oximeter (NONIN Medical Inc., Plymouth, MN, USA) was used to measure SpO_2_ (%) and HR (bpm) from the left index finger of the subject. Average values of SpO_2_ and HR in each phase were obtained for each subject. The repeated measures of ANOVA (PASW ver. 18.0) were performed to evaluate the differences in SpO_2_ and HR between two oxygen concentrations (21% vs. 92%) and between control and task phases. We excluded the adaptation phase out of the analysis.

## Results

**Figure 1:**
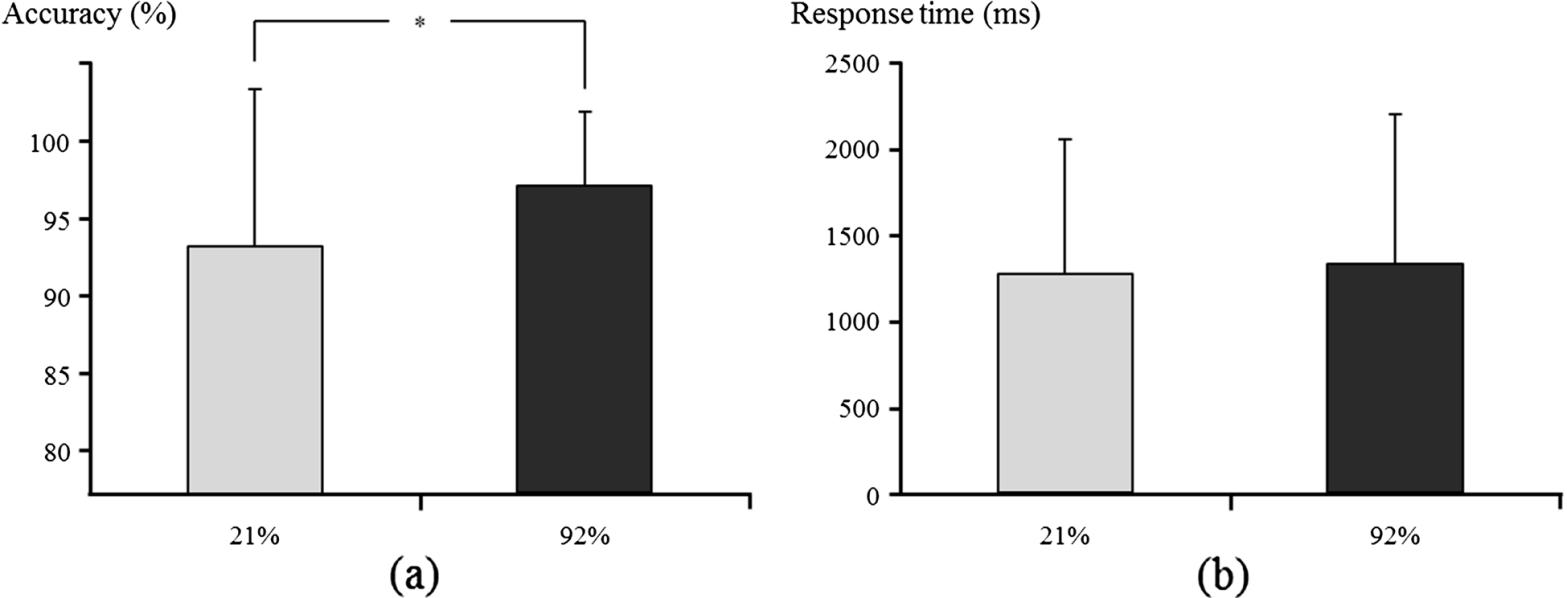
**The 0-back task performance. (a)** Accuracy and **(b)** response time for the administration of 21% and 92% oxygen concentrations.

Figure 1 shows the results of behavioral accuracy and response time during the 0-back task with two different oxygen concentrations. Accuracy on average was 93.2 ± 10.1 for 21% and 97.1 ± 4.7 for 92% oxygen concentration (Figure 1a). The paired *t*-test revealed a significant difference in accuracy between oxygen concentrations (*t* = −2.148, df = 13, *P* = 0.050). Response time on average was 1,275.1 ± 781.1 ms for 21% oxygen and 1,329.6 ± 873.5 ms for 92% oxygen (Figure 1b). Yet, no significant difference in response times was found between oxygen concentrations (*t* = −0.590, df = 13, *P* > 0.05).

**Figure 2:**
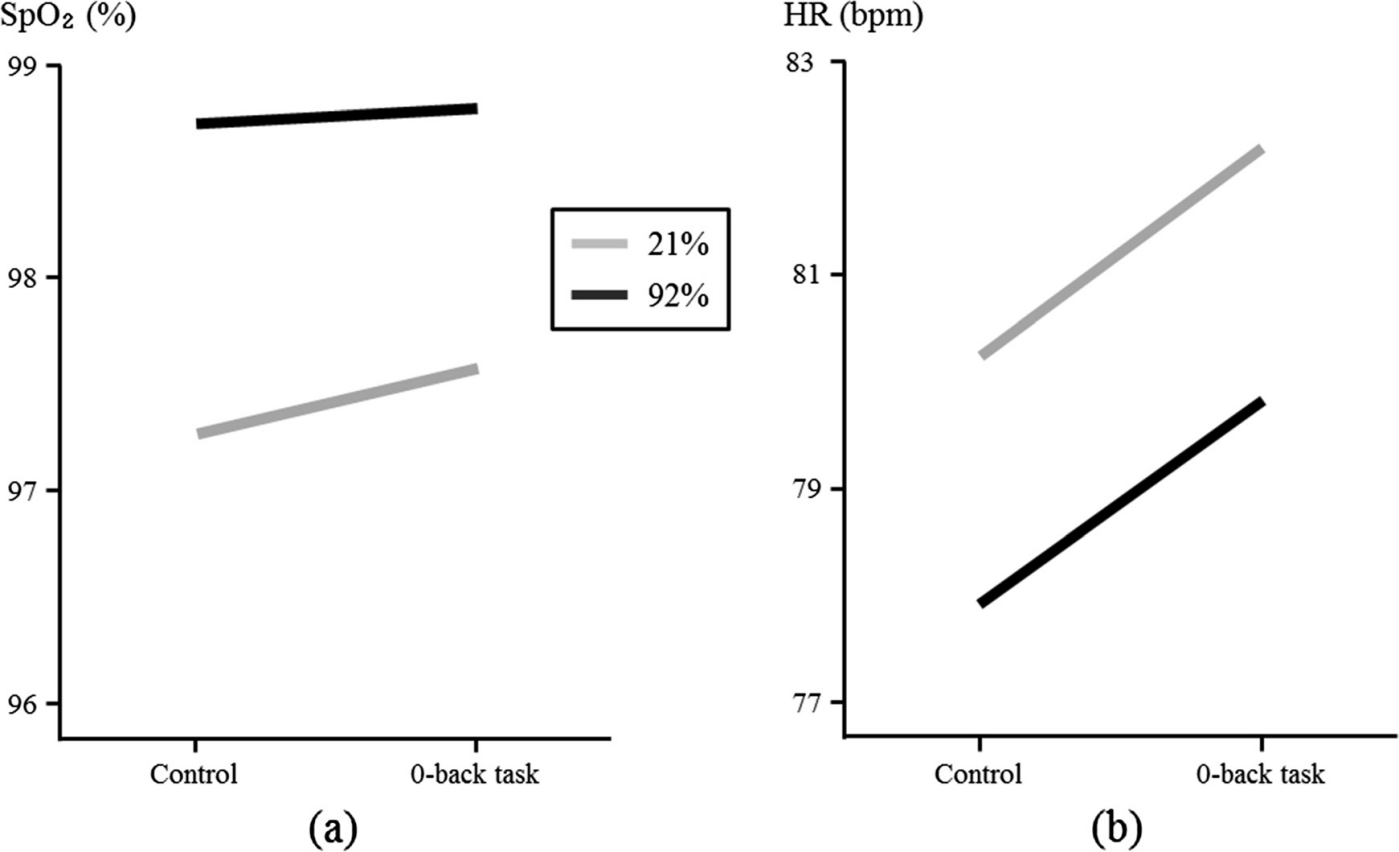
**Physiological responses. (a)** Blood oxygen saturation (SpO_2_) and **(b)** heart rate (HR) with administration of 21% and 92% oxygen concentrations.

Figure 2a shows the average SpO_2_ during control and task phases for 21% and 92% oxygen concentrations. Repeated measures of ANOVA revealed a significant difference in SpO_2_ between oxygen concentrations (*P* < 0.001) and between control and task phases (*P* = 0.018) (Table 1). The administration of 92% oxygen led to greater SpO_2_ than 21% oxygen. Also, greater SpO_2_ was observed during the 0-back task phase than during the control. A significant interaction effect was shown between concentration and phase (*P =* 0.012), indicating a difference in trend of change in SpO_2_ at the two oxygen concentrations.

**Table 1:** **ANOVA results of physiological responses for different conditions**

	**Source**	**Type III sum of squares**	**df**	**Mean square**	***F***	**Sig.**
	Concentration	25.246	1	25.246	189.239	0.000
SpO_2_	Phase	0.521	1	0.521	7.337	0.018
	Concentration × phase	0.191	1	0.191	8.625	0.012
	Concentration	77.002	1	77.002	7.880	0.015
HR	Phase	50.921	1	50.921	23.677	0.000
	Concentration × phase	0.006	1	0.006	0.003	0.959

df, degrees of freedom. F, *F* value. Sig., significant.

The mean HR of each phase (control and task) was shown in Figure 2b according to oxygen concentration. Repeated measures of ANOVA revealed a significant difference in HR between oxygen concentrations (*P* = 0.015) as well as between control and task phases (*P* < 0.001) (Table 1). HR with 92% concentration was less than that with 21% concentration while HR was greater in the task phase than that in the control. No interaction effect was found between concentration and phase (*P* > 0.05).

## Discussion

In the present study, we examined whether high-concentration (92%) oxygen supply changed short-term memory performance, blood oxygen saturation, and heart rate in intellectually and developmentally disabled people.

Previous studies investigated the contribution of high-concentration oxygen supply to cognitive performance in healthy young and elderly individuals [8-20]. Especially, Kim *et al*. (2013) reported the improvement of visual matching task performance owing to high-concentration oxygen supply in people with intellectual and developmental cognitive deficit. The present study extended this finding by showing the improvement of 0-back task performance; an increase in accuracy was observed during exposure to the higher-concentration of oxygen. Therefore, it can be concluded that enriched oxygen can positively affect, at least in the short-term, the working memory of those with intellectual and developmental disability.

Presently, increased SpO_2_ was evident in the presence of 92% oxygen in comparison with 21% oxygen. It is consistent with a view that supply of higher-concentration oxygen increased the blood oxygen saturation level and consequently provided more oxygen to the brain, which in turn resulted in better performance of the 0-back task that requires active brain metabolism. Neural substrates engaged in the 0-back task may benefit from increased circulation of blood oxygen [13,15]. It is well understood that an increase in the supply of metabolic fuel, such as glucose, enhances the production of adenosine triphosphate (ATP) when cognitive demand is high. Such increases in ATP production can facilitate information processing involved in cognitive functions. These improvements of information processing may be reflected by enhancement of cognitive tasks [11]. To metabolize the fuel, the brain requires higher blood oxygen levels. Therefore, strong coupling between cognitive functions and the brain metabolism supports why a transient increase in oxygen concentration levels would be helpful.

In the present study, SpO_2_ during the 0-back task phase (cognitive processing phase) was increased compared with that during the control phase. This agrees with the results of previous studies [8,11,13,16-18,22,23], where oxygen requirements increased during cognitive processing. There was an interaction effect between oxygen concentration and phase because of the lower increase rate of SpO_2_ from control phase to 0-back task phase at 92% oxygen compared with 21% oxygen. This may be because sufficient oxygen is already being supplied for cognitive processing at the control phase when 92% oxygen is administered.

Presently, there was a decrease in HR at 92% oxygen compared with 21% oxygen. Hyperoxia can reduce the resting heart rate [24]. Therefore, the present decrease in HR was likely due to hyperoxia [8-10,14,16-18]. HR during the 0-back task phase was increased compared with that during the control phase. This is consistent with the view that the cardiac load increases during cognitive processing, as has been reported [8,9,14,16,18].

In conclusion, the administration of 92% oxygen increased 0-back task performance of intellectually and developmentally disabled people, in association with increased SpO_2_ and decreased HR. The results are an objective and reliable demonstration of the positive effects of high-concentration oxygen supply on working memory in people with intellectual and developmental deficits, obtained during task performance and by recording changes in physiological signals.

The number of participants in the present study is not sufficiently large. Therefore, it would be necessary though to perform further studies with more participants to translate our results into clinical applications. Various cognitive abilities of intellectually and developmentally disabled people, such as information processing, attention, and verbal abilities, must be studied. Further studies need to be performed to examine the long-term and short-term effects of enriched oxygen on cognitive processing. Moreover, only two bio-signals - SpO_2_ and heart rate - were observed to investigate the impact of high oxygen concentration on the body. However, in order to comprehensively and specifically analyze the effects of high oxygen concentration in terms of physiological anthropology, additional studies are required. These are based on various assessment methods, such as functional magnetic resonance imaging, electroencephalography, magnetoencephalography, blood flow, blood pressure, and heart rate variability; these can measure the response of the central nervous system and the peripheral nervous system in the brain and the whole body system.
